# *Six1* haploinsufficiency is associated with activation of NF-κB and TNF-related transcriptional signatures in aging mice

**DOI:** 10.1038/s41419-026-08831-w

**Published:** 2026-05-06

**Authors:** Tianxu Guo, Han Liu, Junjun Ma, Huanyu Yan, Yanglin Chen, Lihua Zhao, Xiyun Guo, Limin Lv, Yixi Wang, Linxin Cheng, Guang Yang, Yu Zhang, Jinbo Yu, Xi Wang, Shuguang Duo, Xihe Li, Rongfeng Li

**Affiliations:** 1 Jiangsu Provincial Key Laboratory of Biological Therapy for Organ Failure, Nanjing Medical University, Nanjing, 211166 China; 2 State Key Laboratory of Reproductive Medicine and Offspring Health, Center for Global Health, School of Public Health, Nanjing Medical University, Nanjing, 211166 China; 3 The State Key Laboratory of Reproductive Regulation and Breeding of Grassland Livestock, Inner Mongolia University, Hohhot, 010070 China; 4 National Center of Technology Innovation for Dairy, Hohhot, 010020 China; 5 Laboratory Animal Center, Institute of Zoology, Chinese Academy of Sciences, Beijing, 100101 China; 6 Inner Mongolia Saikexing Institute of Breeding and Reproductive Biotechnology in Domestic Animal, Hohhot, 011517 China; 7 Key Laboratory of Targeted Intervention of Cardiovascular Disease, Collaborative Innovation Center for Cardiovascular Disease Translational Medicine, Nanjing Medical University, Nanjing, 210006 China

**Keywords:** Senescence, Transcriptomics, RNAi, Transcriptional regulatory elements

## Abstract

The *Six1* (*SIX* homeobox 1) gene is pivotal in renal and pulmonary development and differentiation. Its dysregulation is implicated in oncogenesis and tumor progression via enhancing cell proliferation and delaying senescence. However, whether or how it functions in the natural aging have not been investigated. To answer this question, we generated *Six1* gene knockout mice using CRISPR-Cas9 technology. All *Six1* biallelic knockout mice died at birth since the underdeveloped lungs. In *Six1*^*+/–*^ mice, the developmental deficiencies in kidneys with vacuolar degeneration and epithelial disruption in renal tubules, as well as hematopoietic interstitial infiltration and lungs with interstitial condensation and alveolar hypoplasia were observed. These developmental deficiencies persist with age and age-dependent phenotypes become more pronounced in *Six1*^*+/–*^ mice compared to the wild-type, with upregulation of senescence markers (p16, p53) and senescence-associated secreted factors (e.g., TNF-α, TIMP-2), increased α-SMA expression and collagen deposition, as well as susceptibility to pulmonary fibrosis. Transcriptomic sequencing coupled with bioinformatics analysis indicated that genes with altered expression in *Six1*^*+/–*^ mouse lungs showed enrichment in pathways associated with senescence, including the NF-κB and TNF signaling pathways. These transcriptional patterns were also associated with gene sets involved in mitochondrial metabolic processes. Collectively, these findings suggest that *Six1* haploinsufficiency is associated with transcriptional signatures linked to aging-related pathways under physiological conditions in mice, providing potential clues for future studies exploring mechanisms of aging.

## Introduction

*Six1*, a member of the SIX homology gene family, is crucial for the development and differentiation of several organs, particularly the kidney and lung. In the lung, *Six1* contributes to branching morphogenesis and epithelial differentiation [1], while during kidney development, it is expressed in the posterior renal mesenchyme, essential for glomerulus and nephron formation [2]. Its expression markedly organ/tissue specific declines in adulthood, yet abnormal overexpression has been associated with enhanced proliferation and metastasis in multiple cancers, including breast, colorectal, and hepatocellular carcinoma [3]. Nevertheless, the *Six1* instant activation alleviates the renal ischemia/reperfusion injury by promoting proliferation and suppressing inflammation [4]. The above researches suggest that the appropriate spatio-temporal expression of *Six1* is requisite for organism development and cell proliferation, any abnormality will result in severe disorder.

Cellular senescence is a complex biological process characterized by irreversible cell-cycle arrest [5], metabolic alterations [6], and the secretion of pro-inflammatory factors [7] collectively known as the senescence-associated secretory phenotype (SASP). It contributes to aging-related decline and diseases such as cancer, cardiovascular disorders, and neurodegeneration [8]. Because senescence markers differ across cell types, multiple indicators including p16INK4A [9], p21 [10], p15 [11], p53 [12], SA-β-gal [13], and DNA damage markers [14] are required for the comprehensive in vivo evaluation. At the organismal level, senescence is closely related to mitochondrial dysfunction [15], oxidative stress [16], and chronic inflammation[17], reflecting an interplay between genetic and metabolic stress responses. At the molecular level, aging is orchestrated by key regulators such as p53–p21 [11, 12] and p16INK4a–Rb pathways [9] that mediate cell-cycle arrest, while SIRT1 [18], FOXO3 [19], and Klotho [20] maintain metabolic and longevity programs[21]. Inflammatory pathways, especially NF-κB and TNF signaling [12, 22], drive SASP formation and link chronic inflammation to tissue degeneration. The perturbations in mitochondrial dynamics, NAD⁺ metabolism, and the AMPK/mTOR axis further reinforce these aging processes [23, 24].

Within this biological framework, developmental transcription factors that also regulate stress and inflammatory signaling may play dual roles in both organogenesis and senescence. Six1could represent one such factor since it is known to modulate proliferation and differentiation through inhibiting the non-canonical NF-κB and p53 signaling pathways and protecting cells from senescence and inflammatory death [12]. However, its role in natural aging and systemic senescence remains unclear. Establishing the Six1 deficiency models followed by mechanistic investigation may therefore illuminate how developmental regulators influence tissue maintenance, inflammatory signaling, and the molecular transition from homeostasis to senescence.

To explore the underlying mechanisms for senescence, the diverse animal models (e.g., C57BL/6, BALB/c, SAMP8[25], Ercc1^–^^/–^[26], and LmnaG609G[27]) and cell models (e.g., WI-38[28] and IMR-90 fibroblasts[29]) have been established and achieved abundant and valuable progresses. In this study, we generated *Six1* knockout mice via CRISPR/Cas9-mediated genome editing to examine the effects of *Six1* deficiency on kidney and lung development and its role in the aging process. We hypothesize that *Six1* exerts a protective effect against age-associated degeneration, and that its loss accelerates senescence, particularly in tissues with high *Six1* expression. This study may provide new insights into the mechanisms linking the developmental genes to aging regulation and the potential strategies to mitigate age-related dysfunction.

## Results

### The developmental deficiency of kidney and lung in *Six1* gene knockout mice

**Fig. 1:**
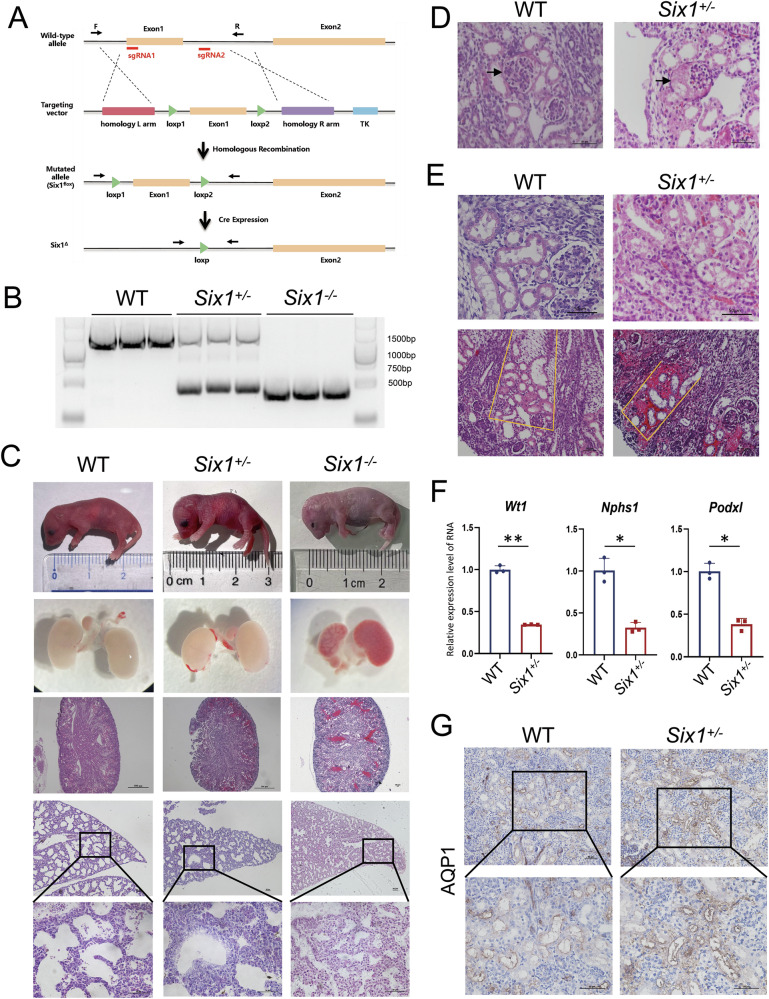
The developmental deficiency of *Six1* gene knockout mice. **A** Pattern of mouse *Six1* gene knockout by CRISPR-Cas9. **B** Electropherogram of PCR amplification products for *Six1* knockout identification. **C** Comparison of the appearance of newborn *Six1*^+/–^ mice, *Six1*^*–/–*^ mice and wild-type mice, and HE comparison of kidneys and lungs. **D**, **E** HE staining of neonatal mouse kidneys, the arrow points to the expansion of Bowman’s cyst cavity. **F** RT-qPCR detects mRNA expression levels of glomerular-specific markers *Wt1*, *Podxl*, and *Nphs1*. **G** Immunohistochemical precise localization of AQP1 expression.

We generated a *Six1* knockout mouse model using CRISPR/Cas9 and zygote pronuclear injection techniques (Fig. 1A and Supplementary Fig. 1A). Neonatal *Six1*^–/–^ mice died at birth, while *Six1*^+/–^ and wild-type mice survived. A total of 14 *Six1*^*+/–*^ genotype mice and 12 wild-type mice were used in this study (Fig. 1B).

Autopsies revealed that *Six1*^–/–^ mice had smaller kidneys with hematopoietic interstitial infiltration, and underdeveloped lungs with interstitial condensation and alveolar hypoplasia (Fig. 1C). Lymphocyte infiltration was increased in their livers, but no cardiac or splenic abnormalities were observed (Supplementary Fig. 1B).

Renal comparison between *Six1*^+/–^ and wild-type mice showed vacuolar degeneration and epithelial disruption in renal tubules of *Six1*^+/–^ mice, with hematopoietic interstitial infiltration (Fig. 1D). Bowman’s capsule lumen was dilated, and podocyte markers WT1, PODXL, and NPHS1 were downregulated, indicating renal papillae dysplasia (Fig. 1E, F). AQP1 positivity in disrupted tubules confirmed proximal tubule dysplasia in *Six1*^+/–^ mice. No significant abnormalities were observed in the lungs of *Six1*^+/–^ mice compared to wild-type mice at birth (Fig. 1G).

### The developmental deficiency in *Six1*^+/–^ mice is not repaired with age

**Fig. 2:**
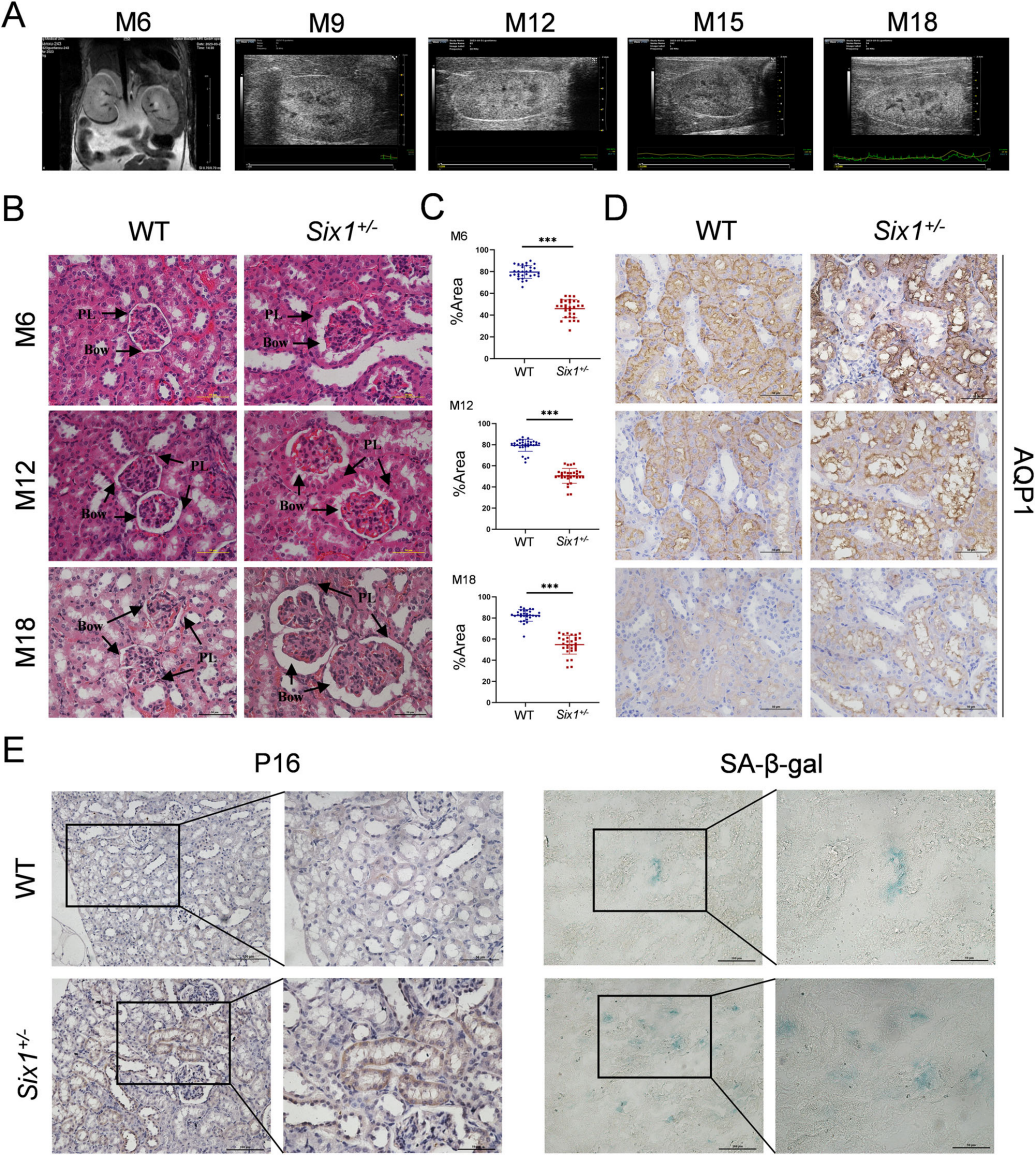
The developmental deficiency is not repaired. **A** Representative MRI/B ultrasound maps of *Six1*^+/–^ mice at different ages. **B** Representative diagrams of HE staining of glomeruli in WT/*Six1*^+/–^ mice at different ages (PL: single-layer flat epithelial-capillary wall layer, Bow: Bowman’s capsule lumen; the length of the scale bar in the diagram is 50 μm. **C** Statistical analysis of the ratio of glomerular to renal corpuscle area. Three groups of three mice each with 10 randomly selected glomeruli were analyzed statistically. **D** Immunohistochemical staining analysis of the proximal tubule-specific marker AQP1. **E** Expression of p16 in the kidneys of 18-month-old mice identified by immunohistochemistry; SA-β-gal staining of frozen sections of kidneys from 18-month-old mice.

We paid attention to investigate whether renal developmental deficiency in *Six1*^+/–^ mice is repaired with age. NMR/B ultrasound examinations of *Six1*^+/-^ mice at 6, 8, 12, 15, and 18 months excluded kidney stones, renal tumors, renal cysts, and hydronephrosis, indicating no increased susceptibility to these renal diseases due to the single *Six1* allele knockout (Fig. 2A).

Dissections of 6-, 12-, and 18-month-old *Six1*^+/–^ mice revealed abnormal thickening of the renal capsule wall, consisting of a single flattened epithelial layer compared to wild-type controls (Fig. 2B). Quantitative analysis of glomerular to renal corpuscle area ratios revealed a significantly larger Bowman’s capsule lumen area in *Six1*^+/-^ mice (Fig. 2C). The thickened epithelium of the renal vesicle wall may progress to form crescents. Vacuolar degeneration and epithelial cell fragmentation and detachment in renal tubules persisted from 6 to 18 months in *Six1*^+/–^ mice. Immunohistochemical staining identified AQP1-positive proximal tubule breaks (Fig. 2D), suggesting that renal developmental damage in *Six1*^+/-^ mice is not resolved with age.

### The aging of kidneys is exacerbated in *Six1*^+/–^ mice

**Fig. 3:**
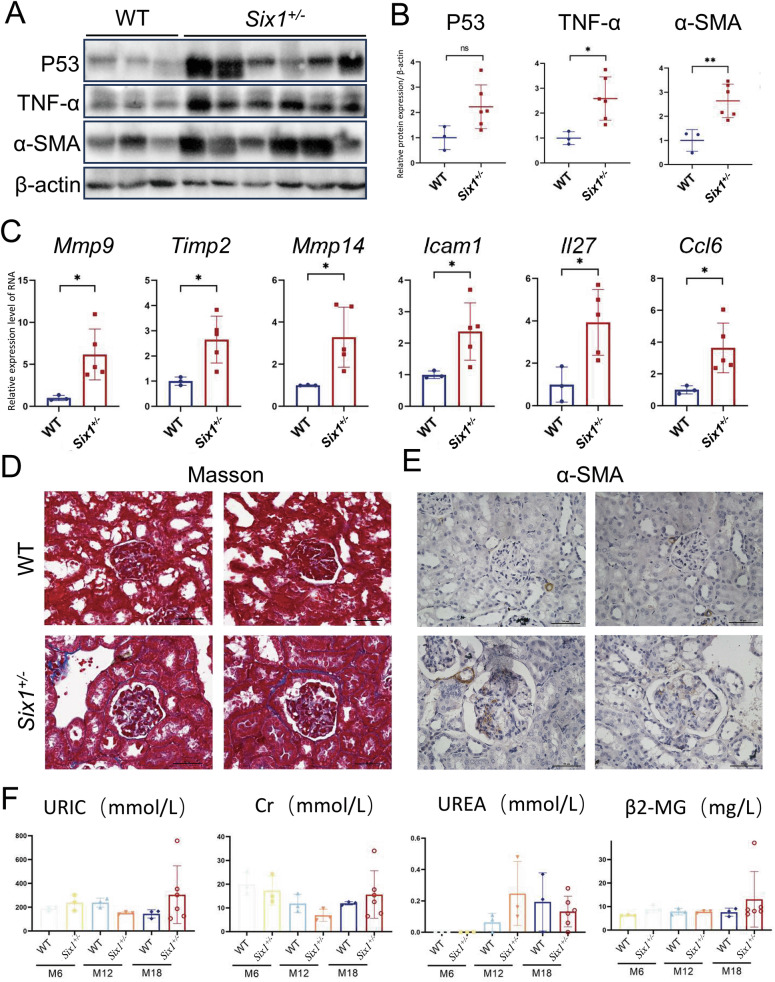
The aging of kidneys is exacerbated. **A** Western blot analysis of classical aging markers (p53, TNF-α, and α-SMA) in the kidneys of 18-month-old mice. **B** Quantification of the protein levels of p53, TNF-α, and α-SMA in the kidneys of 18-month-old mice. Protein expression was normalized to β-actin (**C**) RT-qPCR results of SASP in kidneys of 18-month-old mice. **D** Masson staining of kidneys from 18-month-old mice. **E** Immunohistochemical detection of α-SMA expression in kidneys of 18-month-old. **F** Serum biochemical assays of renal function in mice of both genotypes at 6, 12, and 18 months of age.

We assessed aging markers in 12-month-old *Six1*^+/–^ mice and observed no significant exacerbation of renal aging phenotypes. By 18 months, mice enter a stage of aging, then we tried to compare the aging severity in *Six1*^+/–^ versus wild-type mice of the same age. Immunohistochemical staining for p16 in 18-month-old mice revealed increased expression in the *Six1*^+/–^ group (Fig. 2E). SA-β-gal activity, a marker of cellular senescence, was also elevated in the kidneys of *Six1*^+/–^ mice as assessed by staining and activity assays (Fig. 2E). Additionally, p53, a key senescence marker in the DNA damage response, was significantly upregulated in the kidneys of 18-month-old *Six1*^+/–^ mice compared to wild-type controls (Fig. 3A).

Aging impairs immune clearance of senescent cells, prompting the secretion of senescence-associated secretory phenotype (SASP), which accumulate and hasten aging. In 18-month-old *Six1*^+/–^ mice, we observed upregulated SASP mRNA and TNF-α expression in kidneys (Fig. 3B, C), along with increased inflammatory cell infiltration (Supplementary Fig. 1C), suggesting an exacerbated aging phenotype.

As mice age, renal fibrosis typically increases. Masson staining of kidneys from 18-month-old wild-type and *Six1*^+/–^ mice revealed no significant collagen deposition in either group (Fig. 3D). However, α-SMA expression was upregulated in the kidneys of *Six1*^*+/–*^ mice, not observed in wild-type glomeruli (Fig. 3E), indicating an early towards glomerular fibrosis.

For the renal function examination, serum samples from 6-, 12-, and 18-month-old wild-type and *Six1*^+/–^ mice were collected and renal function markers: urea, creatinine (Cr), uric acid (URIC), and β-micro-globulin (β2-MG) were analyzed. Creatinine, a muscle metabolism product, did not show significant differences between groups, indicating there is no significant decrease in glomerular filtration rate. β2-MG, which should accumulate when the renal detoxification is impaired, was not significantly elevated in the *Six1*^+/–^ group (Fig. 3F). However, the elevated levels of urea and URIC, products of protein and nucleic acid metabolism respectively were observed in the 18-month-old *Six1*^+/–^ mice (Fig. 3F), indicating that *Six1* monoallelic knockout exacerbated aging in mice characterized by glomerular filtration damage.

### The aging of lung is exacerbated in *Six1*^+/–^ mice

**Fig. 4:**
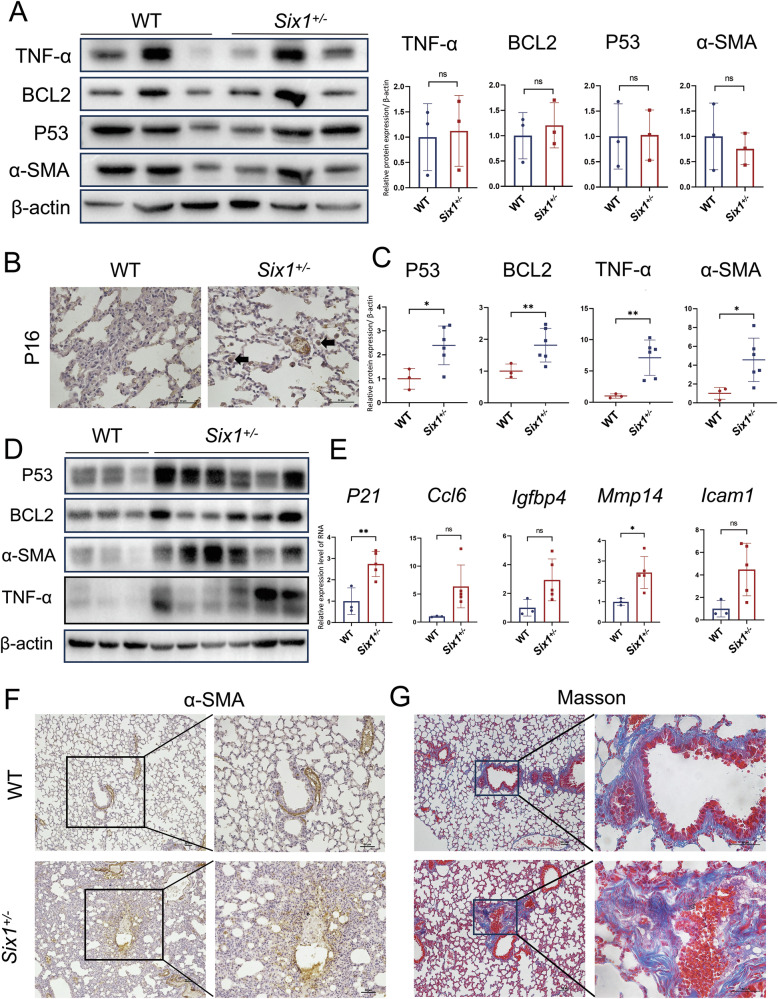
The aging of lung is exacerbated. **A** Western blot identification and quantification of gray value of lung senescence-related markers in 12-month-old mice, biological replicates, *n* = 3. **B** Expression of P16 in the lungs of 18-month-old mice identified by immunohistochemistry. **C** Quantification of protein levels of classical aging markers (P53, BCL2, TNF-α, and α-SMA) in the lungs of 18-month-old mice. Protein expression was normalized to β-actin. **D** Western blot analysis of classical aging markers (P53, BCL2, TNF-α, and α-SMA) in the lungs of 18-month-old mice. **E** Results of RT-qPCR performed for SASP in the lungs of 18-month-old mice. **F** Immunohistochemical staining for the fibrosis marker α-SMA in the lungs of 18-month-old mice. **G** Representative image of Masson staining in 18-month-old mouse lungs.

Aging markers in 12-month-old *Six1*^+/–^ mice showed no significant lung aging phenotype (Fig. 4A), likely due to the young age and minimal aging-related processes. This indicates that a single *Six1* allele knockout does not induce premature aging. The senescence phenotype observed in 18-month-old *Six1*^+/–^ mice likely accumulated post-12 months. With age, lung elasticity declines and airway resistance increases in mice. Morphological analysis revealed enlarged alveoli in 12- and 18-month-old mice, more so in *Six1*^+/–^ mice (Supplementary Fig. 1D), suggesting increased loss of lung elasticity in this group.

Aging markers in the lungs of *Six1*^+/–^ mice mirrored those in the kidneys, with increased P16, P53, TNF-α, and BCL2 expression (Fig. 4B–D), suggesting potential resistance to apoptosis. The majority of *Six1*^+/–^ mice also showed upregulated SASP mRNA levels in the lungs (Fig. 4E).

Lung fibrosis was identified in 18-month-old mice, with α-SMA expression and abnormal staining in *Six1*^+/–^ mice suggesting regional fibrotic aggregates (Fig. 4F). Masson staining revealed increased collagen fiber deposition in *Six1*^*+/–*^ lungs (Fig. 4G), indicating an age-associated fibrotic trend.

### The abnormally expressed genes are enriched in the senescence-related NF-κB and TNF signaling pathways

**Fig. 5:**
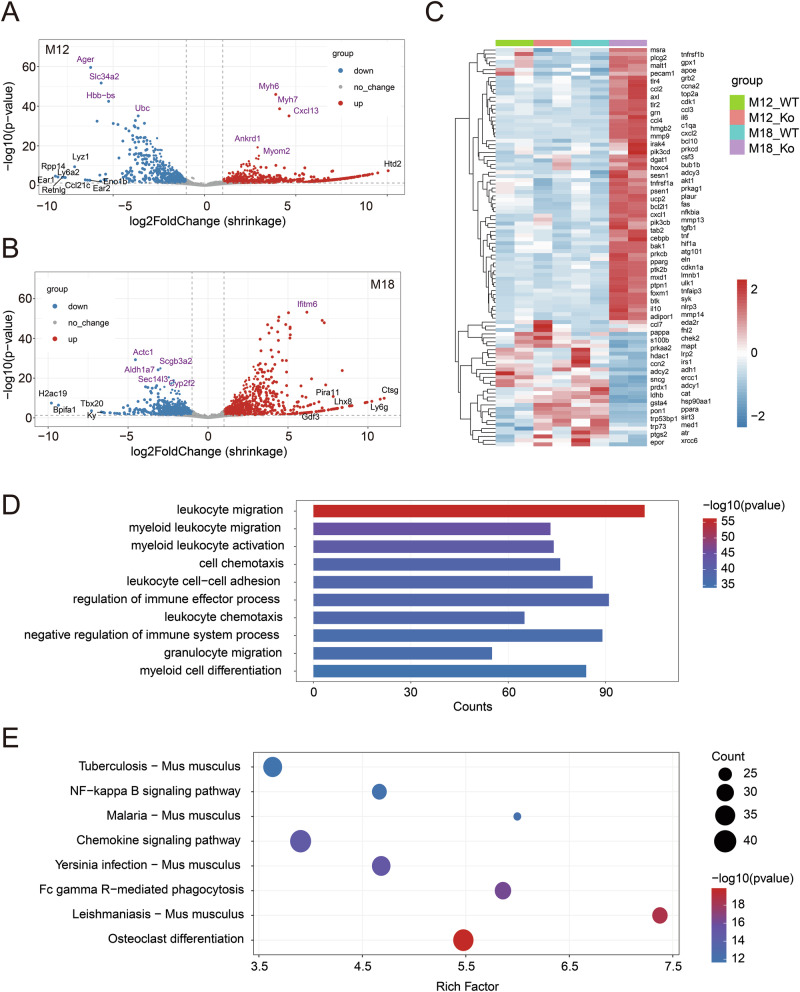
RNA-seq analysis of the lung tissue in 12- and 18-month-old mice. **A** Transcriptome sequencing volcano plot of candidate differentially expressed genes in 12-month-old mouse lungs (*Six1*^+/–^ group vs wild-type group ; *n* = 2 biological replicates per group). **B** Transcriptome sequencing volcano plot of candidate differentially expressed genes in 18-month-old mouse lungs (*Six1*^+/–^ group vs wild-type group ; *n* = 2 biological replicates per group). **C** Heatmap analysis of the expression levels of senescence-related genes in the lungs of *Six1*^+/–^ mice vs wild-type mice. **D** GO enrichment analysis of genes showing increased expression trends in 18-month-old mouse lung transcriptome sequencing in *Six1*^+/–^ group vs wild-type group. **E** Bubble plot of KEGG enrichment analysis of genes showing increased expression trends in 18-month-old mouse lung transcriptome sequencing, *Six1*^+/–^ group vs wild-type group.

Since in adult wild-type mice, *Six1* is expressed in the lung but not in the kidney and its expression is significantly reduced in the lung of 18-month-old *Six1*^*+/–*^ mice (Supplementary Fig. 2A), we chose lung tissue for RNA-seq. (Supplementary Fig. 2A). We performed exploratory transcriptome sequencing of lung tissues from 12- and 18-month-old mice. In 12-month-old mice, 1029 genes were upregulated and 841 genes were downregulated (Fig. 5A), whereas in 18-month-old *Six1*^*+/–*^ mice, 1130 genes were upregulated and 1122 genes were downregulated (Fig. 5B). Using Aging Atlas, a multi-omics senescence database, we identified 101 differential genes related to senescence in the 18-month-old group. The heatmap comparison showed increased expression of senescence-associated genes in the lungs of *Six1*^+/–^ mice (Fig. 5C).

To elucidate the functions of candidate differentially expressed genes, we conducted GO and KEGG enrichment analyses on upregulated genes in *Six1*^+/–^ mice compared to wild-types. GO biological process enrichment analysis suggested that the upregulated genes may be involved in innate immunity and inflammatory regulation, including leukocyte migration, myeloid leukocyte migration and activation, cell and leukocyte chemotaxis, leukocyte cell-cell adhesion, and regulation of immune effector process (Fig. 5D). These findings indicated that *Six1* haploinsufficiency exacerbated the inflammaging phenotype in mouse lungs, which was characterized by abnormal activation and chemotactic infiltration of myeloid immune cells—well-established hallmarks of the aging process.

KEGG pathway enrichment analysis showed that the upregulated genes were significantly enriched in core inflammatory regulatory pathways including the NF-kappa B signaling pathway and chemokine signaling pathway (Fig. 5E). Among these pathways, the upregulated expression of core genes in the NF-kappa B signaling pathway is consistent with age-associated changes in pulmonary inflammation, was further validated by our western blot and qPCR results (Fig. 4C–E). The consistent findings from GO and KEGG analyses collectively suggested that *Six1* haploinsufficiency drives age-associated pulmonary inflammatory and senescence-related changes in mice, mainly through modulating innate immune activation and inflammatory signaling pathways.

The transcripts of *Six2* and *Six4* were not detected in the RNA-seq data, and qPCR analysis confirmed that neither *Six2* nor *Six4* was compensatorily upregulated (Supplementary Fig. 2B). In addition, the expression changes of senescence-associated marker genes showed in the RNA-seq data were consistent with those tested by qPCR (Supplementary Fig. 2C, Fig. 4E). These qPCR results supported the reliability of the sequencing data.

### Mitochondrial metabolic pathway transcripts are downregulated in the lungs of *Six1*^+/–^ mice

**Fig. 6:**
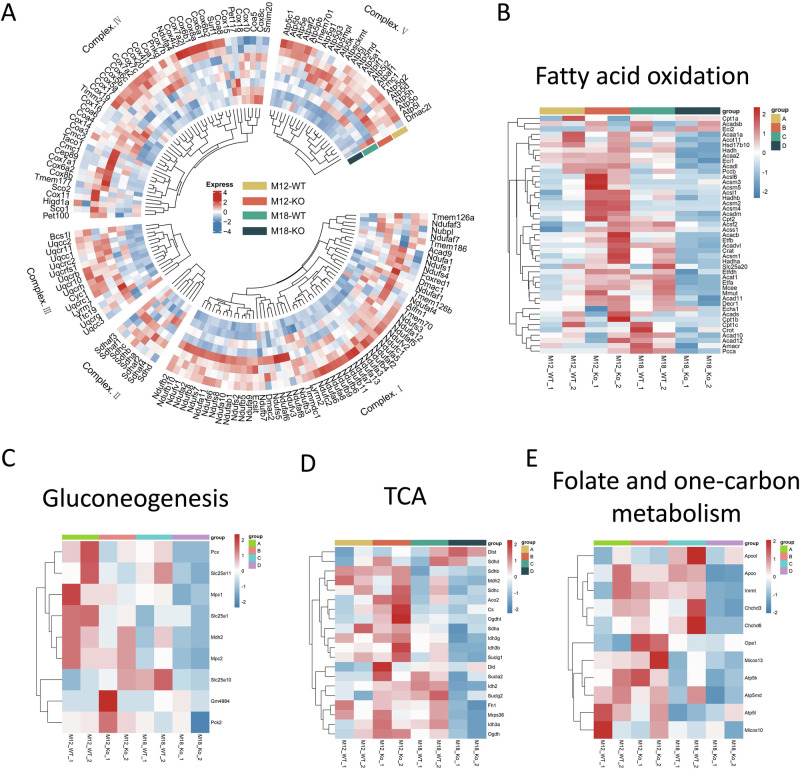
Expression patterns of mitochondrial metabolic pathway genes in the lungs. **A** Heatmap of mitochondrial complex-related gene expression in 12-month-old and 18-month-old mice (RNA-seq dataset; *n* = 2 biological replicates per group). **B** Expression of fatty acid oxidation-related genes in mouse lungs. **C** Expression of gluconeogenesis-related genes in mouse lungs. **D** Expression of tricarboxylic acid cycle-related genes in mouse lungs. **E** Expression of genes related to folate and one-carbon metabolism in mouse lungs.

We examined mitochondrial complex-related gene expression in 12- and 18-month-old *Six1*^+/–^ mice, using MitoCarta3.0. The analysis revealed a general downregulation of these genes in 18-month-old *Six1*^+/–^ mice, including factors for complex I-V assembly (Fig. 6A). These complexes are crucial for oxidative phosphorylation (OXPHOS) and ATP formation, and their dysfunction can lead to mitochondrial impairment. By contrast, 12-month-old *Six1*^+/–^ mice exhibited relatively preserved expression of these genes (Fig. 6A), suggesting that the decline becomes more evident with advanced age.

Further analysis of metabolic pathways in the lungs of 18-month-old *Six1*^+/–^ mice revealed downregulation of transcripts associated with fatty acid oxidation, the tricarboxylic acid (TCA) cycle, and gluconeogenesis (Fig. 6B–D), folate and one-carbon metabolism not significantly affected (Fig. 6E). These findings suggest that *Six1* haploinsufficiency is associated with transcript-level downregulation of mitochondrial metabolic pathways, which may reflect age-related molecular changes in the lungs.

## Discussion

Cellular senescence has complex effects on the organism. Accumulation of senescent cells during aging is generally deleterious, particularly in the absence of senescence-suppressing genes during embryonic development, which may lead to premature cellular senescence, tissue and organ dysplasia, or even defects; conversely, senescence following tissue damage is beneficial for the clearance of damaged cells, especially in specific disease contexts where cellular senescence is indispensable. The *Six1* gene, which functions as both a transcriptional activator and a transcriptional repressor [30], plays a key role in regulating embryonic cell development and organ differentiation. It is critical for the expansion of progenitor cells in the early stages of development. The biallelic knockdown of *Six1* impairs the development of kidney [31], lung [32], inner ear [33], and muscle [34] to varying degrees, which may be due to the inhibition of the progenitor cell proliferation by its mutation [35]. In our study, *Six1*^*–/–*^ mice died at birth, and *Six1*^*+/–*^ mice survived, which is consistent with the results in the study on *Six1*^*–/–*^ mice mentioned above, but they mainly focused on the development of the inner ear and muscles, and did not make in-depth exploration of other organs. When searching for the main cause of perinatal lethality in *Six1*^*–/–*^ mice, we found that the death may be related to the dysplasia of the kidney and lung.

*Six1*^*+/–*^ mice survived, but exhibited renal dysplasia, including impaired glomerular and proximal tubular development, and with age, we found that renal defects in *Six1*^*+/*–^ mice at birth were not repaired, and that these abnormalities persisted into adulthood and were associated with an aging phenotype. Upregulation of senescence markers (p16, p53) and senescence-associated secreted factors (e.g., TNF-α, TIMP-2) in *Six1*^+/–^ mice compared to wild type suggests accelerated senescence of kidneys and lungs in these mice at later stages of survival. Masson staining results, upregulation of α-SMA expression, and increase in collagen deposition in *Six1*^*+/–*^ mice indicate an increased senescence associated with heightened susceptibility to fibrosis, particularly in the lungs. This is consistent with the known role of *Six1* in organogenesis [36, 37]. This suggests that *Six1* may play a protective role in maintaining tissue homeostasis and delaying the ageing process, and that *Six1* may have a sustained effect on tissue maintenance and repair mechanisms, which are impaired in the absence of *Six1*. However, considering that *Six1* is essential for kidney and lung development, its knockout will inevitably lead to organ dysfunction and mimic aging [38, 39]. For example, renal insufficiency leads to waste accumulation, metabolic stress, and systemic inflammation, which in turn induce cellular senescence and fibrosis. Pulmonary dysfunction can lead to impaired oxygen exchange, oxidative stress, and fibrosis, which may also mimic aging. Thus, although we detected positive results for multiple aging markers in *Six1*^*+/–*^ mice, these senescence and fibrosis markers that we observed may be secondary to chronic organ stress rather than true accelerated aging. Therefore, in concluding that *Six1* deletion can induce accelerated kidney and lung aging in mice, the possibility that it is a secondary effect of organ dysfunction cannot be completely discarded from discussion.

One of the most notable beneficial effects of senescence is tumor limitation, as the senescence of cells can prevent the proliferation and expansion of precancerous cells. The role of *Six1* is context-dependent. It is typically silenced post-development but reactivated in numerous cancers, where it promotes proliferation, metastasis, and immune evasion[40, 41]. This functional duality underscores the complexity of *Six1* signaling. In our transcriptome analysis of mouse lungs, enrichment analyses showed upregulation of genes involved in inflammation, senescence, and NF-κB pathways in *Six1*^*+/–*^ mice, providing transcriptional indications of heightened SASPs and altered mitochondrial OXPHOS-related gene expression[42, 43], which suggests that a monoallelic knockout of *Six1* may disrupt inflammatory homeostasis and exacerbate lung inflammation and senescence. These findings are consistent with the established role of *Six1* in regulating cellular senescence and influencing non-classical NF-κB signaling pathways [44, 45]. Hyperactivation of P53, a known inducer of cellular damage and senescence, was also observed in the context of *Six1* deficiency, suggesting a possible link between *Six1* and p53-mediated senescence pathways [46, 47]. Given the dual role of *Six1* in senescence and tumorigenesis, future studies must delve into the specific mechanisms of *Six1* action and how to precisely regulate its expression for therapeutic purposes. On the one hand, enhancing the function of *Six1* may help to slow down the aging process and improve age-related organ dysfunction. On the other hand, inhibition of *Six1* activity may be beneficial for the treatment of certain types of tumors especially in those cases where *Six1* overexpression is closely associated with tumor progression. Future studies are needed to finely regulate *Six1* activity at the molecular level for clinical applications in ageing retardation and tumor treatment [48, 49].

In the present study, we included preliminary kidney function assessments, which revealed impaired glomerular and tubular development, persistent structural abnormalities, and increased fibrosis in *Six1*^*+/−*^ mice. These results provide initial evidence supporting the impact of *Six1* haploinsufficiency on renal physiology and age-associated tissue changes. Functional analyses of other organs, including the lung and skeletal muscle, were not performed in the current study, since we originally focused on age-related metabolic and transcriptional alterations. We performed RNA-seq analysis on lung tissue, and observed the changes in mitochondrial-related gene expression. The molecular findings provide indirect evidence of mitochondrial dysfunction rather than the direct functional validation. Future studies incorporating the direct physiological assays—such as pulmonary function tests, muscle performance evaluations and lifespan analyses will be essential to comprehensively characterize the systemic impact of *Six1* deficiency on natural aging. Rescuing *Six1* expression in postnatal *Six1*^*+/−*^ pups will help determine whether the observed renal and pulmonary aging phenotypes are truly age-related or secondary to developmental defects.

In conclusion, we demonstrate that *Six1* haploinsufficiency causes persistent developmental defects that evolve into an aging-like state in key organs. Our work establishes a link between *Six1*, chronic inflammation, and tissue senescence, providing a foundation for future research into its precise molecular mechanisms in the aging process.

## Method details

### Animal care and use

All experiments with animals were approved by the Institutional Animal Care and Use Committee (IACUC) of the Institute of Zoology, Chinese Academy of Sciences (CAS). And the corresponding ethical approval code is IOZ-IACUC-2022-002. The methods were carried out in “accordance” with the approved guidelines. The detailed information on the use of all mice is summarized in Supplementary Table 1.

### *Six1* knockout mice production

In this study, the *Six1* gene was conditionally deleted via CRISPR/Cas9 and a loxP-Cre recombinase genetic approach as illustrated in Fig. 1A. Genomic DNA containing *Six1* was cloned from a 129/Sv mouse genomic library and characterized through sequencing analysis. To target the Exon 1 and the Intron between Exon 1 and 2, two single-guide RNAs (m*Six1*-sgRNA1 and m*Six1*-sgRNA2) (Supplementary Figure 1A) were designed using the online CRISPR Design Tool (http://crispr.mit.edu/), and subsequently cloned into the sgRNA expressing vector. The sequence of sgRNAs are as follows: sgRNA1: AACCAGCAGCATCCACCCGG; sgRNA2: TTCGCCGGAGCCAGCCCGGA. The m*Six1*-sgRNA expressing plasmids and the Cas9 expressing plasmid were linearized, transcribed in vitro, and purified following previously established protocols [48], separately. In addition, the targeting vector was constructed by inserting loxP sites at the beginning of Exon 1 and within the intron between Exon 1 and Exon 2.

The targeting vector, m*Six1*-sgRNAs and Cas9 expressing plasmids were co-microinjected into one-cell zygotes of in vivo recovery, and the injected zygotes were transferred into recipient females via oviduct transfer to generate *Six1*^+/fl^ mice. The heterozygous F1 progeny were intercrossed to generate *Six1*^fl/fl^ mice. The female *Six1*^+/fl^ and *Six1*^fl/fl^ mice and male Cre mice were used for oocytes and sperm cryopreservation, respectively. In vitro fertilization (IVF) was performed, and the resulting IVF embryos were transferred into recipients. The genotypes of neonatal mice were analyzed to identify *Six1*^–/–^ and *Six1*^*+/–*^ mice. The gender of mice was not considered as a biological variable.

### Genotyping by PCR

Mouse tail samples were subjected to thorough dissociation using magnetic beads. Total genomic DNA (gDNA) was obtained using TIAN-amp Genomic DNA kit (TIANGEN). Oligonucleotide Primers used in PCR and their amplified product size were from gDNA. Genotyping polymerase chain reaction primers used were as follows: Forward primer,5′-CACCCTTTTCAGCACCCCAGT-3′; Reverse primer,5′-GACTCAGACCAGCTTCAAGACGG AGCGAAAGA-3′.

### Real-time quantitative PCR analysis

Total RNA was isolated from the frozen tissues using RNAiso Plus (total RNA extraction reagent; Takara Bio). For the cells, total RNA was extracted using the Total RNA Kit (Promega) according to the manufacturer’s instructions, Reverse transcription was performed 1 μg of total RNA using HisScriptⅡ One Step RT-PCR Kit (Vazyme Biotech, Nanjing, China), and real-time quantitative PCR was performed using ChamQ^tm^SYBRqPCR Master Mix (Vazyme Biotech) in 7500 Real-time System (Applied Biosystems, Waltham, MA, USA). β-actin was used as internal control. All samples were assayed in triplicate. The sequences of the primer pairs are shown in Supplementary Table 2.

### Western blotting

Whole kidney or lung specimens were collected from different groups: 18-month-old control group (*n* = 3) and experimental group (*n* = 6), 12-month-old control group (*n* = 3) and experimental group (*n* = 6). Tissues were homogenized and lysed in radioimmunoprecipitation (RIPA) buffer supplemented with protease inhibitor cocktail. Protein concentrations were determined, and equal amounts of protein from each sample were resolved on SDS-polyacrylamide gels and then transferred onto PVDF membranes. The membranes were blocked with 5% nonfat milk in TBST at room temperature for 2 h, followed by incubation with primary antibodies at 4 C overnight and then with the corresponding secondary antibodies at room temperature for 1 h. Following another series of washes with TBST, the immunoreactive bands were visualized using an enhanced chemiluminescence (ECL) substrate and detected with a ChemiDoc XRS+ gel documentation system (Bio-Rad Laboratories, Hercules, CA, USA). The signal intensity of protein bands was quantified using ImageJ software (National Institutes of Health, USA). Full-length, uncropped Western blots are shown in **Supplementary_WB_Raw_All.pdf**. Antibody-related information is shown in the Supplementary Table 3.

### Histology and Immunohistochemistry (IHC)

Formalin-fixed paraffin-embedded tissue sections(5 μm) were deparaffinized in xylene (2 times for 20 min each) and hydrated using 100% ethanol (twice for 5 min), 90% ethanol (5 min), 80% ethanol (5 min), 75% ethanol (5 min) and distilled water (twice for 3 min each). The sections were conducted for Hematoxylin Eosin staining by standard protocol.

For IHC staining, Paraffin sections were deparaffinized and rehydrated in water. Followed by blocking of endogenous peroxidase (3% H_2_O_2_). Antigen retrieval was done by heating sections to 121 °C in citrate buffer for 3 min. The slides allowed to cool down for 30 min and were then rinsed in TBS three times for 5 min. then blocked with Normal Goat Serum for 1 h at room temperature followed by primary antibody incubation overnight at 4 °C. After washing, sections were incubated with horseradish peroxidase (HRP)-conjugated secondary antibody at room temperature for 1 h, washed and processed using 3,3-Diaminobenzidine tetrahydrochloride. Counterstaining was carried out with hematoxylin. Antibody-related information is shown in the Supplementary Table 3.

### SA-β-gal activity staining

To detect senescence-associated SA-β-gal activity, cryosections of mouse kidneys were processed by using Senescence β-Galactosidase Staining Kit (C0602, Beyotime), the sections and cells were photographed and analyzed under a fluorescence microscope.

### Masson staining

Kidney and lung tissues were fixed in 10% neutral buffered, embedded in paraffin and the paraffin slices were sequentially placed in gradient xylene solution and ethanol solution and then washed with water. The slices were soaked overnight in potassium dichromate and then rinsed under running water. After hematoxylin iron staining, the samples were washed with running water and the slices were placed in Ponceau red acid fuchsin for 3 min. After soaking in phosphomolybdate brine solution for 2 min, the paraffin sections were placed in aniline blue dye solution for 3 min and dyed. It was differentiated with 1% glacial acetic acid for a few seconds and dehydrated with anhydrous ethanol. Finally, the slices were soaked in xylene and sealed with a neutral resin.

### Assessment of renal function in serum

After the mice fasted for 24 h, the eyeballs were extracted and blood was collected. We prepared 800 μL whole blood for each sample and left it for 3–4 h to wait for the blood clot to precipitate. After centrifugation at 1000 × *g*–2000 × *g* for 10 min, light yellow serum was found. The serum was transferred to in a new Eppendorf, the dead cells were removed by centrifugation at 3000 × *g* again for 10 min. Finally, serum samples were obtained by centrifugation at a low temperature of 10,000 × *g* for 20 min.

URCA/ Cr / URIC /β2-MG were measured by an automatic biochemical analyzer.

### B-ultrasound detection

The mice fasted and forbade water for 24 h before B-ultrasound detection. B-ultrasound was tested by Laboratory Animal Center of Nanjing Medical University.

### RNA sequencing (RNA-seq)

Two Six1^⁺/⁻^ and two wild type mice were used at each age (12 and 18 months). RNA-seq libraries following the SMART-seq2 protocol [50]. Briefly, in the first step, each sample was stored in 2 µL lysis buffer (0.2% TritonX-100 and 40 U/mL Recombinant RNase Inhibitor). After incubating the samples in 10 μL Oligo(dT) buffer at 72 °C for 3 min, the tubes were immediately put back on ice for 2 min. Then, 4 µL of 5X RT buffer and 4 µL Reverse Transcriptase buffer (ABclonal, RK20310) were added to each sample for first-strand cDNA synthesis, followed by cDNA amplification using Amplification Module PCR Mix (1 µL PCR primer and 29 µL PCR Master Mix) for 16 cycles. Thereafter, the library was purified with 1X DNA Clean beads (Vazyme, N411-01). Tn5 Enzyme mix and PCR mix (ABclonal, RK20237) were used for tagmentation to construct sequencing libraries from the amplified cDNA. Finally, the DNA library amplification was performed by adding 25 µL PCR Mix and 5 µL amplification primers (10 µM) to the products, followed by library purification with 0.6X and 0.15X DNA Clean beads (Vazyme, N411-01). The quality of the libraries for sequencing was verified with PerkinElmer LabChip GX Touch. Sequencing was performed on the Illumina NovaSeq 6000 platform in the PE150 mode.

### RNA-seq data preprocessing

The RNA-seq data were first subjected to adaptor trimming and low-quality read filtering with flexbar (version 2.5) [51] with the following parameters: -u 6 -m 36 -ae RIGHT -at 2 -ao 2 -x 3 -y 1. The trimmed paired-end reads were then aligned to the mouse reference genome (mm10) by STAR (version 2.7.10b) [51] under the parameters: -outFilterMismatchNoverLmax 0.1 --alignIntronMin 20 --alignIntronMax 1000000 --alignSJoverhangMin 6 --alignSJDBoverhangMin 1 --outFilterType BySJout --outFilterIntronMotifs RemoveNoncanonicalUnannotated, followed by gene expression quantification by HTSeq (v0.13.5) [52]. Fragments per kilobase of transcript per million fragments mapped (FPKM) values of genes were calculated as ${{FPKM}}_{g}=\frac{{C}_{g}}{{l}_{g}{\sum }_{i}{C}_{i}}\times {10}^{9}$, where Cg denotes the fragment count of gene g, and Lg is the total non-redundant exonic length of gene g.

### RNA-seq data analysis

Differential expression analysis was performed using the DESeq2 R package (v1.30.1). DESeq2 models RNA-seq count data using a negative binomial distribution with gene-wise dispersion estimation.Given the limited number of biological replicates (*n* = 2 per group), the analysis was considered exploratory and hypothesis-generating. Genes meeting an adjusted p-value (FDR) < 0.05 and |log2 fold change | > 1 were identified as candidate differentially expressed genes. No definitive transcriptome-wide inference is claimed. To obtain more stable estimates of expression changes, log2 fold change shrinkage was applied using the lfcShrink function with the *apeglm* method implemented in DESeq2.

Functional enrichment analysis Functional enrichment analysis was performed for genes showing increased or decreased expression trends using the clusterProfiler R package (v4.0.5) for Gene Ontology (GO) biological process terms and Kyoto Encyclopedia of Genes and Genomes (KEGG) pathways. An FDR < 0.05 was used as a reference threshold; however, given the limited sample size, enrichment results were interpreted cautiously and considered descriptive rather than definitive.

To place our findings within a broader aging research context, we obtained a set of aging-associated genes reported in the Aging Atlas database (https://ngdc.cncb.ac.cn/aging/index). The expression levels of these genes were then examined in our RNA-seq dataset. Gene expression values were normalized using FPKM (Fragments Per Kilobase of transcript per Million mapped reads) and log2-transformed for comparative analysis of expression patterns. All statistical analyses and visualizations were performed in the R environment (v4.0.3).

### The statistic analysis

All data are presented as mean ± standard error of the mean (SEM). Statistical analyses were performed using GraphPad Prism 9 (San Diego, CA, USA). For comparisons between two groups, a paired Student’s *t* test was applied assuming equal variance, as variance between groups was not formally tested. p values of less than 0.05, 0.01, 0.001, or 0.0001 were considered statistically significant and are indicated in the figures as *, **, ***, or ****, respectively.

### Sample size, inclusion/exclusion criteria and randomization

No statistical methods were used to predetermine sample size. Sample sizes were based on commonly accepted standards in the field. In total, twelve *Six1*^*+/−*^ mice were generated using the described breeding strategy. Among them, three pups were collected at birth for early postnatal phenotypic assessment. Of the remaining animals, three *Six1*^*+/−*^ mice were euthanized and analyzed at 12 months of age, and six mice were dissected at 18 months of age for aging-related phenotypic and molecular analyses. These cohorts were used for all downstream histological, molecular, and physiological experiments. No samples or animals were excluded from the analysis; therefore, no inclusion or exclusion criteria were applied. No method of randomization was used to assign animals to experimental groups, as group allocation was determined solely by age.

## Supplementary information

Supplementary Information

Supplement table 1

Supplementary Table 2

Supplementary Table 3

Supplementary Figures 3A, 4A, 4D, 2A

## Data Availability

Raw sequencing reads have been deposited in the Sequence Read Archive (SRA) and are accessible through the NCBI BioProject database under the accession PRJNA1406166.

## References

[1] Guo L, Li F, Liu H, Kong D, Chen C, Sun S. SIX1 amplification modulates stemness and tumorigenesis in breast cancer. J Transl Med. 2023;21:866. 38031089 10.1186/s12967-023-04679-2 PMC10685563

[2] Wang CA, Jedlicka P, Patrick AN, Micalizzi DS, Lemmer KC, Deitsch E, et al. SIX1 induces lymphangiogenesis and metastasis via upregulation of VEGF-C in mouse models of breast cancer. J Clin Invest. 2012;122:1895–906. 22466647 10.1172/JCI59858 PMC3336979

[3] Correction to “SIX1 Is Upregulated in Gastric Cancer and Regulates Proliferation and Invasion by Targeting the ERK Pathway and Promoting Epithelial-Mesenchymal Transition”. Cell Biochem Funct. 2025;43:e70045. 10.1002/cbf.70045 39817567

[4] Jin Y, Zhang M, Li M, Zhang H, Zhao L, Qian C, et al. SIX1 activation is involved in cell proliferation, migration, and anti-inflammation of acute ischemia/reperfusion injury in mice. Front Mol Biosci. 2021;8:725319. 34513929 10.3389/fmolb.2021.725319 PMC8427868

[5] Demaria M, O’Leary MN, Chang J, Shao L, Liu S, Alimirah F, et al. Cellular senescence promotes adverse effects of chemotherapy and cancer relapse. Cancer Discov. 2017;7:165–76. 27979832 10.1158/2159-8290.CD-16-0241 PMC5296251

[6] Bussian TJ, Aziz A, Meyer CF, Swenson BL, van Deursen JM, Baker DJ. Clearance of senescent glial cells prevents tau-dependent pathology and cognitive decline. Nature. 2018;562:578–82. 30232451 10.1038/s41586-018-0543-y PMC6206507

[7] Schafer MJ, White TA, Iijima K, Haak AJ, Ligresti G, Atkinson EJ, et al. Cellular senescence mediates fibrotic pulmonary disease. Nat Commun. 2017;8:14532. 28230051 10.1038/ncomms14532 PMC5331226

[8] Childs BG, Gluscevic M, Baker DJ, Laberge RM, Marquess D, Dananberg J, et al. Senescent cells: an emerging target for diseases of ageing. Nat Rev Drug Discov. 2017;16:718–35. 28729727 10.1038/nrd.2017.116 PMC5942225

[9] De Lope C, Martín-Alonso S, Auzmendi-Iriarte J, Escudero C, Mulet I, Larrasa-Alonso J, et al. SIX1 represses senescence and promotes SOX2-mediated cellular plasticity during tumorigenesis. Sci Rep. 2019;9:1412. 30723235 10.1038/s41598-018-38176-0 PMC6363751

[10] de Lope C, García-Lucena R, Magariños M, León Y, Casa-Rodríguez N, Contreras N, et al. Dysfunction of programmed embryo senescence is linked to genetic developmental defects. Development. 2023;150:dev200903. 10.1242/dev.200903 PMC10259514 37017267

[11] Towers CG, Guarnieri AL, Micalizzi DS, Harrell JC, Gillen AE, Kim J, et al. The Six1 oncoprotein downregulates p53 via concomitant regulation of RPL26 and microRNA-27a-3p. Nat Commun. 2015;6:10077. 26687066 10.1038/ncomms10077 PMC4703841

[12] Adrados I, Larrasa-Alonso J, Galarreta A, López-Antona I, Menéndez C, Abad M, et al. The homeoprotein SIX1 controls cellular senescence through the regulation of p16INK4A and differentiation-related genes. Oncogene. 2016;35:3485–94. 26500063 10.1038/onc.2015.408 PMC5730042

[13] Birch J, Gil J. Senescence and the SASP: many therapeutic avenues. Genes Dev. 2020;34:1565–76. 33262144 10.1101/gad.343129.120 PMC7706700

[14] Gorgoulis V, Adams PD, Alimonti A, Bennett DC, Bischof O, Bishop C, et al. Cellular senescence: defining a path forward. Cell. 2019;179:813–27. 31675495 10.1016/j.cell.2019.10.005

[15] Calcinotto A, Kohli J, Zagato E, Pellegrini L, Demaria M, Alimonti A. Cellular senescence: aging, cancer, and injury. Physiol Rev. 2019;99:1047–78. 30648461 10.1152/physrev.00020.2018

[16] Borghesan M, Hoogaars WMH, Varela-Eirin M, Talma N, Demaria M. A senescence-centric view of aging: implications for longevity and disease. Trends Cell Biol. 2020;30:777–91. 32800659 10.1016/j.tcb.2020.07.002

[17] Ferrucci L, Fabbri E. Inflammageing: chronic inflammation in ageing, cardiovascular disease, and frailty. Nat Rev Cardiol. 2018;15:505–22. 30065258 10.1038/s41569-018-0064-2 PMC6146930

[18] Chen C, Zhou M, Ge Y, Wang X. SIRT1 and aging related signaling pathways. Mech Ageing Dev. 2020;187:111215. 32084459 10.1016/j.mad.2020.111215

[19] Zhao Y, Liu YS. Longevity factor FOXO3: a key regulator in aging-related vascular diseases. Front Cardiovasc Med. 2021;8:778674. 35004893 10.3389/fcvm.2021.778674 PMC8733402

[20] Buchanan S, Combet E, Stenvinkel P, Shiels PGKlotho. Aging, and the Failing Kidney. Front Endocrinol (Lausanne). 2020;11:560. 32982966 10.3389/fendo.2020.00560 PMC7481361

[21] de Gonzalo-Calvo D, Neitzert K, Fernández M, Vega-Naredo I, Caballero B, García-Macía M, et al. Differential inflammatory responses in aging and disease: TNF-alpha and IL-6 as possible biomarkers. Free Radic Biol Med. 2010;49:733–7. 20639132 10.1016/j.freeradbiomed.2010.05.019

[22] Ogrodnik M, Carlos Acosta J, Adams PD, d’Adda di Fagagna F, Baker DJ, Bishop CL, et al. Guidelines for minimal information on cellular senescence experimentation in vivo. Cell. 2024;187:4150–75. 39121846 10.1016/j.cell.2024.05.059 PMC11790242

[23] Li L, Liang Y, Kang L, Liu Y, Gao S, Chen S, et al. Transcriptional regulation of the Warburg effect in cancer by SIX1. Cancer Cell. 2018;33:368–85.e7. 29455928 10.1016/j.ccell.2018.01.010

[24] Song Q, Hou Y, Zhang Y, Liu J, Wang Y, Fu J, et al. Integrated multi-omics approach revealed cellular senescence landscape. Nucleic Acids Res. 2022;50:10947–63. 36243980 10.1093/nar/gkac885 PMC9638896

[25] Akiguchi I, Pallàs M, Budka H, Akiyama H, Ueno M, Han J, et al. SAMP8 mice as a neuropathological model of accelerated brain aging and dementia: Toshio Takeda’s legacy and future directions. Neuropathology. 2017;37:293–305. 28261874 10.1111/neup.12373

[26] Kim DE, Dollé MET, Vermeij WP, Gyenis A, Vogel K, Hoeijmakers JHJ, et al. Deficiency in the DNA repair protein ERCC1 triggers a link between senescence and apoptosis in human fibroblasts and mouse skin. Aging Cell. 2020;19:e13072. 31737985 10.1111/acel.13072 PMC7059167

[27] Blouin S, Hartmann MA, Fratzl-Zelman N, Messmer P, Whisenant D, Erdos MR, et al. Normal bone matrix mineralization but altered growth plate morphology in the Lmna(G609G/G609G) mouse model of progeria. Aging Dis. 2024;16:3204–18. 39571160 10.14336/AD.2024.1094 PMC12339082

[28] Wolf FI, Torsello A, Covacci V, Fasanella S, Montanari M, Boninsegna A, et al. Oxidative DNA damage as a marker of aging in WI-38 human fibroblasts. Exp Gerontol. 2002;37:647–56. 11909682 10.1016/s0531-5565(02)00005-0

[29] Xu C, Huang X, Tong Y, Feng X, Wang Y, Wang C, et al. Icariin modulates the sirtuin/NF‑κB pathway and exerts anti‑aging effects in human lung fibroblasts. Mol Med Rep. 2020;22:3833–9. 33000191 10.3892/mmr.2020.11458 PMC7533484

[30] Zhang X, Liu X, Du Z, Wei L, Fang H, Dong Q, et al. The loss of heterochromatin is associated with multiscale three-dimensional genome reorganization and aberrant transcription during cellular senescence. Genome Res. 2021;31:1121–35. 34140314 10.1101/gr.275235.121 PMC8256869

[31] Cai Y, Song W, Li J, Jing Y, Liang C, Zhang L, et al. The landscape of aging. Sci China Life Sci. 2022;65:2354–454. 36066811 10.1007/s11427-022-2161-3 PMC9446657

[32] Suryadevara V, Hudgins AD, Rajesh A, Pappalardo A, Karpova A, Dey AK, et al. SenNet recommendations for detecting senescent cells in different tissues. Nat Rev Mol Cell Biol. 2024;25:1001–23. 38831121 10.1038/s41580-024-00738-8 PMC11578798

[33] Willcox BJ, Donlon TA, He Q, Chen R, Grove JS, Yano K, et al. FOXO3A genotype is strongly associated with human longevity. Proc Natl Acad Sci USA. 2008;105:13987–92. 18765803 10.1073/pnas.0801030105 PMC2544566

[34] Xu PX, Zheng W, Huang L, Maire P, Laclef C, Silvius D. Six1 is required for the early organogenesis of mammalian kidney. Development. 2003;130:3085–94. 12783782 10.1242/dev.00536 PMC3872112

[35] Lu K, Reddy R, Berika M, Warburton D, El-Hashash AH. Abrogation of Eya1/Six1 disrupts the saccular phase of lung morphogenesis and causes remodeling. Dev Biol. 2013;382:110–23. 23895934 10.1016/j.ydbio.2013.07.019

[36] Ozaki H, Nakamura K, Funahashi J, Ikeda K, Yamada G, Tokano H, et al. Six1 controls patterning of the mouse otic vesicle. Development. 2004;131:551–62. 14695375 10.1242/dev.00943

[37] Niro C, Demignon J, Vincent S, Liu Y, Giordani J, Sgarioto N, et al. Six1 and Six4 gene expression is necessary to activate the fast-type muscle gene program in the mouse primary myotome. Dev Biol. 2010;338:168–82. 19962975 10.1016/j.ydbio.2009.11.031

[38] Laclef C, Hamard G, Demignon J, Souil E, Houbron C, Maire P. Altered myogenesis in Six1-deficient mice. Development. 2003;130:2239–52. 12668636 10.1242/dev.00440

[39] Wurmser M, Chaverot N, Chaverot N, Madani R, Sakai H, Negroni E, Demignon J, et al. SIX1 and SIX4 homeoproteins regulate PAX7+ progenitor cell properties during fetal epaxial myogenesis. Development. 2020;147:dev192465. 10.1242/dev.185975 32591430

[40] Hughes CJ, Fields KM, Danis EP, Hsu JY, Neelakantan D, Vincent MY, et al. SIX1 and EWS/FLI1 co-regulate an anti-metastatic gene network in Ewing Sarcoma. Nat Commun. 2023;14:4357. 37468459 10.1038/s41467-023-39945-w PMC10356808

[41] Zhu Z, Rong Z, Luo Z, Yu Z, Zhang J, Qiu Z, et al. Circular RNA circNHSL1 promotes gastric cancer progression through the miR-1306-3p/SIX1/vimentin axis. Mol Cancer. 2019;18:126. 31438963 10.1186/s12943-019-1054-7 PMC6704702

[42] Kanigur Sultuybek G, Soydas T, Yenmis G. NF-κB as the mediator of metformin’s effect on ageing and ageing-related diseases. Clin Exp Pharm Physiol. 2019;46:413–22. 10.1111/1440-1681.13073 30754072

[43] Jia M, Agudelo Garcia PA, Ovando-Ricardez JA, Tabib T, Bittar HT, Lafyatis RA, et al. Transcriptional changes of the aging lung. Aging Cell. 2023;22:e13969. 37706427 10.1111/acel.13969 PMC10577555

[44] Gadd S, Huff V, Walz AL, Ooms A, Armstrong AE, Gerhard DS, et al. A Children’s Oncology Group and TARGET initiative exploring the genetic landscape of Wilms tumor. Nat Genet. 2017;49:1487–94. 28825729 10.1038/ng.3940 PMC5712232

[45] Liu Z, Mar KB, Hanners NW, Perelman SS, Kanchwala M, Xing C, et al. A NIK-SIX signalling axis controls inflammation by targeted silencing of non-canonical NF-κB. Nature. 2019;568:249–53. 30894749 10.1038/s41586-019-1041-6 PMC6812682

[46] Wegert J, Ishaque N, Vardapour R, Geörg C, Gu Z, Bieg M, et al. Mutations in the SIX1/2 pathway and the DROSHA/DGCR8 miRNA microprocessor complex underlie high-risk blastemal type Wilms tumors. Cancer Cell. 2015;27:298–311. 25670083 10.1016/j.ccell.2015.01.002

[47] Aging Atlas: a multi-omics database for aging biology. Nucleic Acids Res. 2021;49:D825-d30. 10.1093/nar/gkaa894 PMC7779027 33119753

[48] He X, Memczak S, Qu J, Belmonte JCI, Liu GH. Single-cell omics in ageing: a young and growing field. Nat Metab. 2020;2:293–302. 32694606 10.1038/s42255-020-0196-7

[49] Rizza S, Filomeni G. Denitrosylate and live longer: how ADH5/GSNOR links mitophagy to aging. Autophagy. 2018;14:1285–7. 30029585 10.1080/15548627.2018.1475818 PMC6103690

[50] Picelli S, Faridani OR, Björklund AK, Winberg G, Sagasser S, Sandberg R. Full-length RNA-seq from single cells using Smart-seq2. Nat Protoc. 2014;9:171–81. 24385147 10.1038/nprot.2014.006

[51] Dodt M, Roehr JT, Ahmed R. Dieterich C. FLEXBAR-Flexible Barcode and Adapter Processing for Next-Generation Sequencing Platforms. Biol (Basel). 2012;1:895–905. 10.3390/biology1030895 PMC4009805 24832523

[52] Anders S, Pyl PT, Huber W. HTSeq-a Python framework to work with high-throughput sequencing data. Bioinformatics. 2015;31:166–9. 25260700 10.1093/bioinformatics/btu638 PMC4287950

