# Supplementary material for: Six1 haploinsufficiency is associated with activation of NF-κB and TNF-related transcriptional signatures in aging mice
Source: Cell Death Dis. 2026 May 6;17(1):605. doi: 10.1038/s41419-026-08831-w (PMC13315602; doi:10.1038/s41419-026-08831-w)
Supplement: Supplementary file 1 — Supplementary Information [file 41419_2026_8831_MOESM1_ESM.pdf]

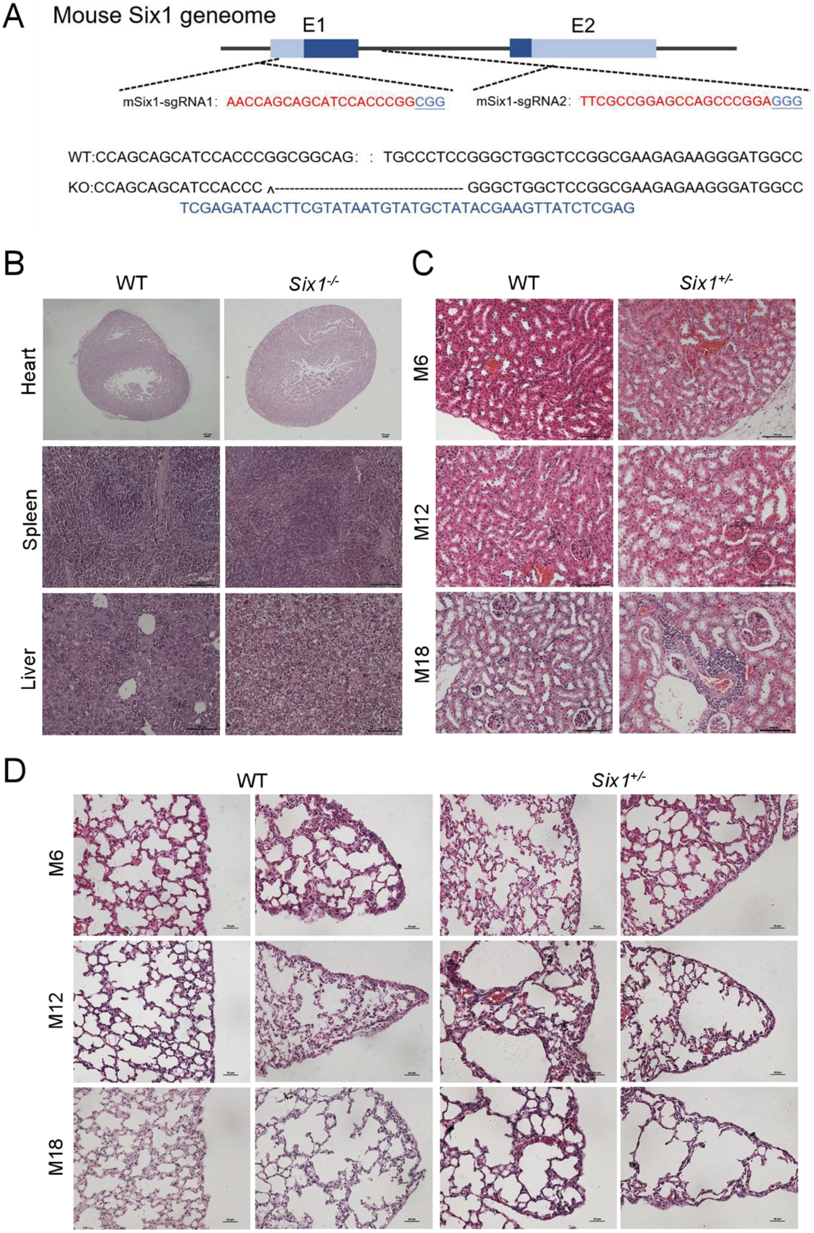

Supplementary Figure 1

Histology of organs in *Six1* mutant mice. **(A)** Target information of mouse *Six1* gene editing. **(B)** Representative images of HE staining of heart, spleen, and liver of newborn *Six1*$^{-/-}$ mice **(C)** Representative diagrams of HE staining of renal tubules in WT and *Six1*$^{+/-}$ mice at different ages. **(D)** HE staining of alveoli near the thoracic wall layer as well as apical alveoli in the lungs of mice of both genotypes at 6, 12 and 18 months of age.

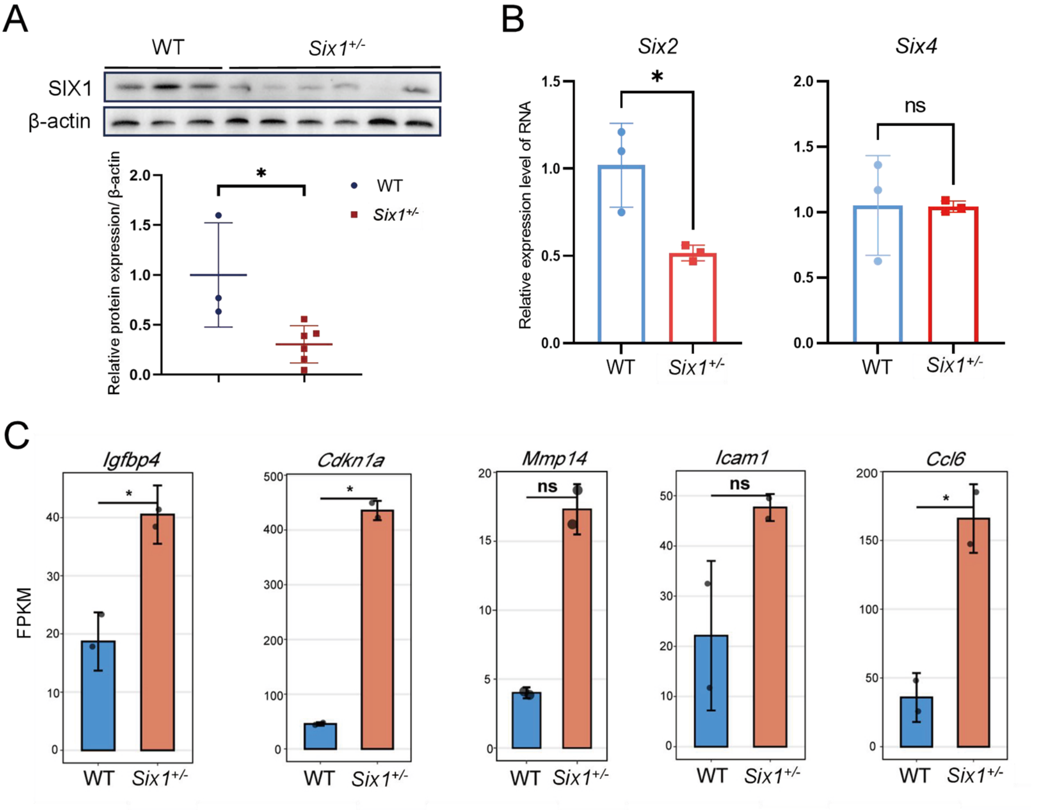

Supplementary Figure 2

Expression of aging-associated markers in 18-month-old mouse lungs. **(A)** Western blot detection of protein expression of classical markers of aging SIX1 in lungs of 18-month-old mice. **(B)** RT-qPCR detects mRNA expression levels of aging-specific markers *Six2* and *Six4* in WT and *Six1*$^{+/-}$ mice at 18 months of ages. **(C)** RNA-seq results of aging-specific markers *Igfbp4*, *Cdkn1a*, *Mmp14*, *Icam1* and *Ccl6* in WT and *Six1*$^{+/-}$ mice at 18 months of ages, with expression values presented as FPKM.
