# Supplementary material for: Six1 haploinsufficiency is associated with activation of NF-κB and TNF-related transcriptional signatures in aging mice
Source: Cell Death Dis. 2026 May 6;17(1):605. doi: 10.1038/s41419-026-08831-w (PMC13315602; doi:10.1038/s41419-026-08831-w)
Supplement: Supplementary file 2 — Supplement table 1 [file 41419_2026_8831_MOESM2_ESM.docx]

**Genotype Number Age/Organ Experiment**

Six1^+/-^ 33

40

42

54

73

75

82

94

102

105

118

225

242

M18/K M18/L M18/K M18/L M18/K M18/L M12/K M18/K M18/L M6/K M12/K M18/K M18/L M9/K M15/K M12/K M12/L P0/K P0/L P0/K P0/L M18/K M18/L M12/K M12/L M12/K M15/K

tissue sections、Western Blot、Total RNA extraction tissue sections、Western Blot、Total RNA extraction tissue sections、Western Blot、Total RNA extraction tissue sections、Western Blot、Total RNA extraction tissue sections、Western Blot、Total RNA extraction

tissue sections、Western Blot、Total RNA extraction、RNA-seq Ultrasound examination

tissue sections、Western Blot、Total RNA extraction、Ultrasound examination tissue sections、Western Blot、Total RNA extraction

tissue sections Ultrasound examination

tissue sections、Western Blot、Ultrasound examination

tissue sections、Western Blot、Total RNA extraction Ultrasound examination

Ultrasound examination

tissue sections、Western Blot、Total RNA extraction

tissue sections、Western Blot、Total RNA extraction、RNA-seq tissue sections

tissue sections Total RNA extraction

tissue sections

tissue sections、Western Blot、Total RNA extraction tissue sections、Western Blot、Total RNA extraction

tissue sections、Western Blot、Total RNA extraction、Ultrasound examination tissue sections、Western Blot、Total RNA extraction、RNA-seq

Ultrasound examination Ultrasound examination

243

M6/K M12/K M12/L

Magnetic Resonance Imaging

tissue sections、Western Blot、Total RNA extraction tissue sections、Western Blot、Total RNA extraction

Six1^-/-^

WT

103

1

2

3

4

5

6

7

8

9

10

11

12

13

14

P0/K P0/L M18/K M18/L M18/K M18/L M18/K M18/L M18/L M18/L M12/K M12/L M12/K M12/L M12/K M12/L M12/L M12/L P0/K P0/L P0/K

P0/L

tissue sections、Total RNA extraction

tissue sections

tissue sections、Western Blot、Total RNA extraction tissue sections、Western Blot、Total RNA extraction tissue sections、Western Blot、Total RNA extraction tissue sections、Western Blot、Total RNA extraction tissue sections、Western Blot、Total RNA extraction tissue sections、Western Blot、Total RNA extraction RNA-seq

RNA-seq

tissue sections、Western Blot、Total RNA extraction tissue sections、Western Blot、Total RNA extraction tissue sections、Western Blot、Total RNA extraction tissue sections、Western Blot、Total RNA extraction tissue sections、Western Blot、Total RNA extraction tissue sections、Western Blot、Total RNA extraction RNA-seq

RNA-seq

tissue sections tissue sections

Total RNA extraction

Total RNA extraction

**Supplementary Table 1. Summary of biological samples and experimental procedures.**

This table lists all samples from **Six1^+/−^**, **Six1^−/−^**, and **wild-type (WT)** mice, including sample IDs, tissue sources, developmental stages, and assays performed.

Assays include tissue sectioning, Western blot, total RNA extraction, RNA-seq, ultrasound, and MRI.

Abbreviations: **K**, kidney; **L**, lung; **M**, months (e.g., M6, M12, M18); **P0**, postnatal day 0.
