# Supplementary material for: Six1 haploinsufficiency is associated with activation of NF-κB and TNF-related transcriptional signatures in aging mice
Source: Cell Death Dis. 2026 May 6;17(1):605. doi: 10.1038/s41419-026-08831-w (PMC13315602; doi:10.1038/s41419-026-08831-w)
Supplement: Supplementary file 3 — Supplementary Table 2 [file 41419_2026_8831_MOESM3_ESM.docx]

**Supplementary Table 2**. Sequences of primers used for RT-qPCR

| **Name** | **Sequence（5’-3’）** |
| --- | --- |
| β-actin-F | GGCTGTATTCCCCTCCATCG |
| β-actin-M | CCAGTTGGTAACAATGCCATGT |
| NPHS1-F | AAGACGAGGAGGAACTGAA |
| NPHS1-M | AAATCGGACAACAAGACG |
| PODXL-F | TCTATGCCGACCTCCACC |
| PODXL-M | AACTGTCACCGCTCTTGC |
| WT1-F | TCTTCCGAGGCATTCAGGATG |
| WT1-R | TGCACACATGAAAGGACGTTT |
| MMP2-F | CAAGTTCCCCGGCGATGTC |
| MMP2-R | TTCTGGTCAAGGTCACCTGTC |
| MMP9-F | GGACCCGAAGCGGACATTG |
| MMP9-R | CGTCGTCGAAATGGGCATCT |
| MMP14-F | CAGTATGGCTACCTACCTCCAG |
| MMP14-R | GCCTTGCCTGTCACTTGTAAA |
| TIMP2-F | TCAGAGCCAAAGCAGTGAGC |
| TIMP2-R | GCCGTGTAGATAAACTCGATGTC |
| IL27-F | CTGTTGCTGCTACCCTTGCTT |
| IL27-R | CACTCCTGGCAATCGAGATTC |
| CXCL1-F | ACTGCACCCAAACCGAAGTC |
| CXCL1-R | TGGGGACACCTTTTAGCATCTT |
| CCL6-F | AAGAAGATCGTCGCTATAACCCT |
| CCL6-R | GCTTAGGCACCTCTGAACTCTC |
| CCL2-F | TAAAAACCTGGATCGGAACCAAA |
| CCL2-R | GCATTAGCTTCAGATTTACGGGT |
| ICAM1-F | TGCCTCTGAAGCTCGGATATAC |
| ICAM1-R | TCTGTCGAACTCCTCAGTCAC |
| P21-F | CCTGGTGATGTCCGACCTG |
| P21-R | CCATGAGCGCATCGCAATC |

Igfbp4-F Igfbp4-R

**Supplementary Table 2. Sequences of primers used for RT–qPCR.**

This table lists all primer sequences used for RT–qPCR analysis, including gene names and corresponding forward (F) and reverse (R) primers.

AGAAGCCCCTGCGTACATTG TGTCCCCACGATCTTCATCTT
