# Supplementary material for: Six1 haploinsufficiency is associated with activation of NF-κB and TNF-related transcriptional signatures in aging mice
Source: Cell Death Dis. 2026 May 6;17(1):605. doi: 10.1038/s41419-026-08831-w (PMC13315602; doi:10.1038/s41419-026-08831-w)
Supplement: Supplementary file 4 — Supplementary Table 3 [file 41419_2026_8831_MOESM4_ESM.docx]

Antibody of WB

| Name | Company | Catalog Number | Actual Dilution Ratio |
| --- | --- | --- | --- |
| Anti-β-Actin | Abclonal | AC028 | 1:10000 |
| Anti-p53 | Abclonal | A10610 | 1:1000 |
| Anti-Bcl-2 | Abclonal | A19693 | 1:1000 |
| Anti-TNFα | Santa Cruz | sc-52746 | 1:1000 |
| Anti-α-SMA | Abcam | ab5694 | 1:1000 |
| Anti-SIX1 | Cell | D4A8K | 1:2000 |
| HRP Goat Anti-Mouse IgG | Abclonal | AS003 | 1:5000 |
| Goat pAb to Rb IgG（HRP） | Abcam | Ab6721 | 1:10000 |

Antibody of IHC

| Name | Company | Catalog Number | Actual Dilution Ratio |
| --- | --- | --- | --- |
| Anti-AQP1 | Proteintech | 20333-1-AP | 1:100 |
| Anti-α-SMA | Abcam | ab5694 | 1:200 |
| Anti-CDKN2A/p16INK4a | Abcam | Ab108349 | 1:100 |
| Goat pAb to Rb IgG（HRP） | Abcam | ab6721 | 1:1000 |

**Supplementary Table 3. Antibodies used for Western blot and immunohistochemistry.**

This table lists all primary and secondary antibodies used in Western blot (WB) and immunohistochemistry (IHC), including antibody name, supplier, catalog number, and dilution ratio.
