# Supplementary figures and images for: Six1 haploinsufficiency is associated with activation of NF-κB and TNF-related transcriptional signatures in aging mice
Source: Cell Death Dis. 2026 May 6;17(1):605. doi: 10.1038/s41419-026-08831-w (PMC13315602; doi:10.1038/s41419-026-08831-w)

**Figure3.A**

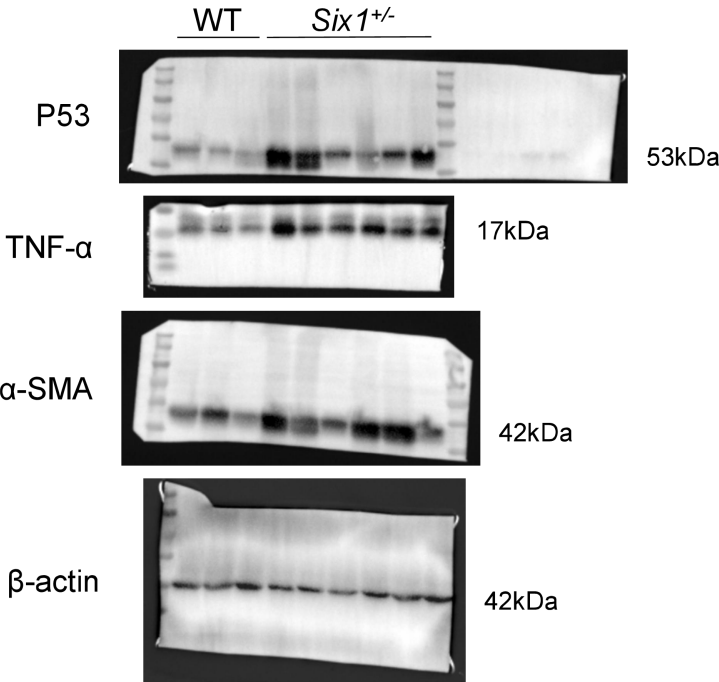

**Figure4.A**

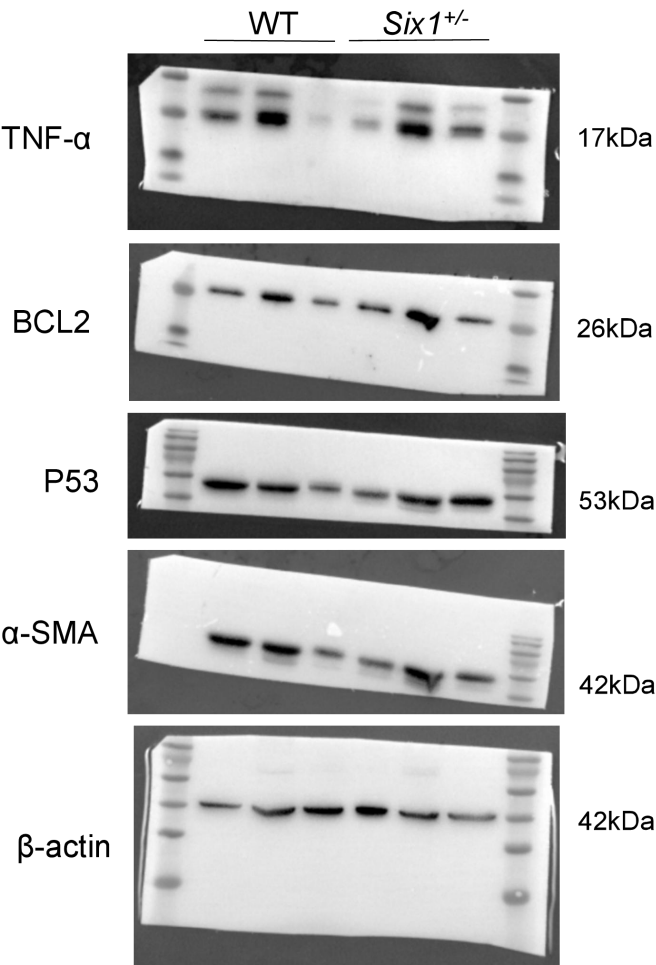

**Figure4.D**

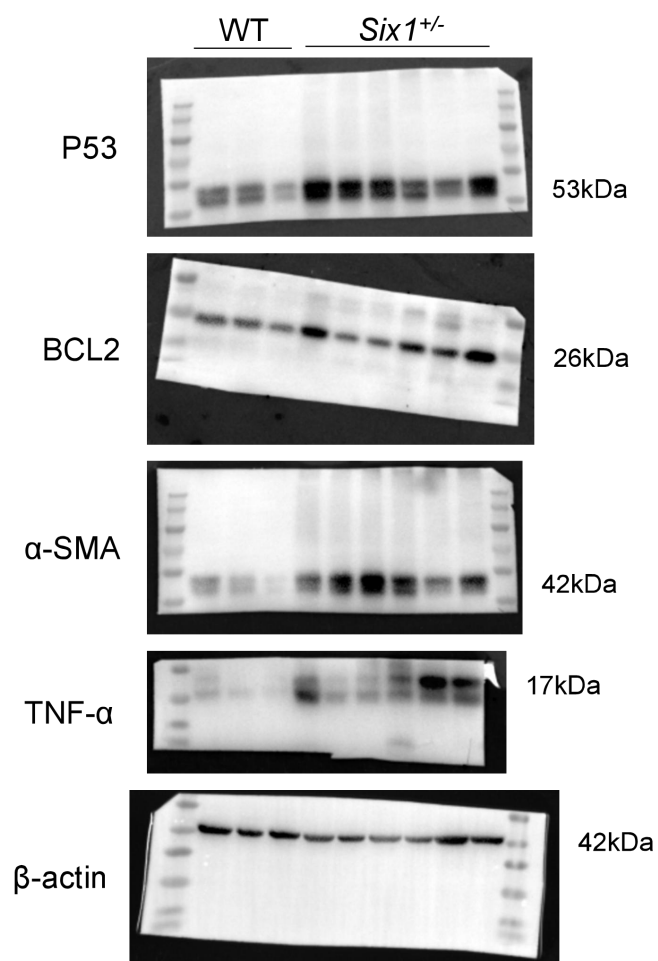

**Supplement Figure 2.A**

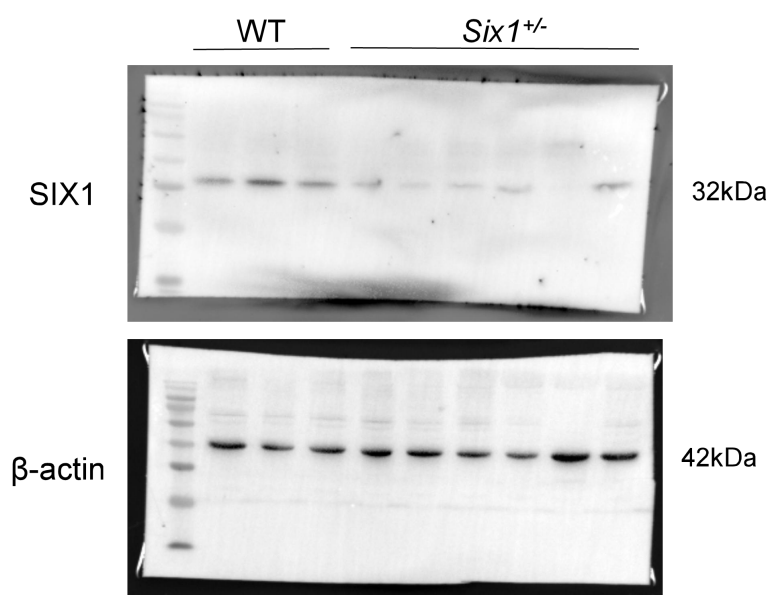

Supplement: Supplementary file 5 — Supplementary Figures 3A, 4A, 4D, 2A [file 41419_2026_8831_MOESM5_ESM.pdf]
